# Allometric equations and carbon stocks assessment for *Bambusa vulgaris* Schrad. ex J.C.Wendl. in the bimodal rainfall forest of Cameroon

**DOI:** 10.1016/j.heliyon.2023.e21251

**Published:** 2023-10-26

**Authors:** Rene Kaam, Martin Tchamba, Barnabas Neba Nfornkah, Cédric Chimi Djomo

**Affiliations:** a Wageningen University & Research, Forest and Nature Conservation Policy Group, P.O. box 47, 6700 AA Wageningen, the Netherlands; b International Bamboo and Rattan Organization (INBAR), Central Africa Regional Office, P.O Box 17056 Yaoundé, Cameroon; c Laboratory of Environmental Geomatics, Department of Forestry, Faculty of Agronomy and Agricultural Sciences, University of Dschang, Dschang, Cameroon, P.O.Box 222-Dschang, Cameroon; d FOKABS - Beside UN Information Center, Tsinga, P.O. Box 181, Yaoundé, Cameroon; e Institute of Agricultural Research for Development (IRAD), Yokadouma, P.O Box. 136, Cameroon

**Keywords:** *Bambusa vulgaris*, Allometric equation, carbon stock, Climate change mitigation, Agroecological zone 5, Cameroon

## Abstract

Over the last two decades, bamboo has received increasing attention owing to its socio-economic and environmental importance. Environmentally, bamboo plays an important role in carbon sequestration, thus enhancing climate change mitigation. In Cameroon, knowledge about the importance of *Bambusa vulgaris* Schrad. ex J.C.Wendl. to climate change mitigation is deficient, despite the fact that it is the most abundant bamboo species in Cameroon's Bimodal rainforest agroecological zone (Agroecological zone 5 - AEZ5). This study was initiated to develop allometric equations and estimate carbon stocks of *B. vulgaris* in Cameroon's AEZ5. The destructive, clump-based method was used for bamboo biomass data collection on 40 clumps and 86 culms. Regression analyses were performed to obtain allometric models for *B. vulgaris* biomass prediction which were used for *B. vulgaris* carbon stocks estimation in AEZ5. The best allometric model for culms was obtained when all predictive variables including age, diameter and height were considered into the model. For clump, the best model was obtained when the number of culms per clump, girth and average diameter were considered in the model. Model quality adjustment was better for clump aboveground biomass (AGB) compared to those of culm AGB. The model of *B. vulgaris* of the evergreen rainfall forest was validated with a bias of 45.5 %. Bamboo aboveground biomass proportions were 77 %, 15 % and 8 %, respectively for culms, branches and leaves. The mean density and carbon stocks of *B. vulgaris* were estimated at 2,0679 culms.ha^−1^, 257 clumps.ha^−1^, and 61.65 tC ha^−1^. *B. vulgaris* has a veritable carbon sequestration capacity which policymakers should consider in climate change mitigation strategies like those linked to payments for ecosystem services, voluntary carbon stocks market, Bonn Challenge, AFR100 initiative, and the Paris agreement ratified by the government of Cameroon.

## 1 Introduction

Bamboo is a woody grass which belongs to the Gramineae family [1]. There are over 1718 different species of bamboo identified in the world [2] although Vorontsova et al. [3] reported 1642 species that can be grouped under three types of bamboo growth forms including the monopodial (running), sympodial (clumping) and the amphipodial bamboo which is a mix of both monopodial and sympodial [1,4]. Bamboo is naturally distributed in tropical, subtropical, and mild temperate zones, and is commonly found in Africa, Asia, Central and South America [[5], [6], [7]]. The total area covered by bamboo forest area is approximately 37 million hectares claiming about 3.2 % of the world's total forest area [2]. Bamboo is an integral part of forests, but it is also widely spread outside forests, including farmlands, riverbanks, roadsides, and urban areas [8].

Over the last two decades, bamboo has received increasing attention for its economic and environmental values. It is a Non-Timber Forest Product (NTFP) which contributes to achieving several environmental services that include biodiversity conservation, soil and water regulation, reclaiming degraded lands, landscape restoration, and climate change mitigation [[5], [6], [7],[9], [10], [11], [12], [13], [14], [15]]. Bamboo plays an important socio-economic role in the sense that it offers opportunities to household, small-scale and medium scale enterprises; it helps meet the population's subsistence and income needs for their livelihood [7,16,17].

In Cameroon, despite the socio-economic and ecological role of bamboo, policies directly regulating this bamboo sector remain weak [13]. Moreover, with the involvement of Cameroon in many initiatives in line with landscape restoration and sustainable resources management including Reducing Emissions from Deforestation and Forest Degradation (REDD+) process, the African Forest Landscape Restoration Initiative (AFR100) which aims to restore 100 million ha of Africa degraded landscape, Bonn Challenge etc., the contribution of bamboo forest appears to be neglected [14]. Considering the fact that deforestation and forest degradation rates continue to increase in Cameroon at the rate of 1.02 % per year [18], afforestation with species like bamboo, taking advantage of its fast-growing rate, becomes one of the countermeasures to mitigate global warming especially in this context where anthropogenic activities are the source of the increasing carbon dioxide in the atmosphere [5,9,14,[19], [20], [21]]. Bamboo is gradually accepted as a restoration tool in Cameroon, for example the 10.13039/100011150Global Environment Facility (GEF) supported Restoration Initiative (TRI) implemented in Cameroon [14].

Forests play and important role in climate change mitigation and global carbon cycle. Several studies have demonstrated the importance of bamboo in Carbon sequestration to fight climate change [6,7,9,15,22,23]. In Cameroon, about 15 bamboo species have been identified [13]. According to bamboo surveys in the five different agroecological zones of Cameroon (AEZs), bamboo covers an area of about 1,215,483 ha with *B. vulgaris* as the most dominant species [9,16]. In the bimodal rainfall forest agroecological zone of Cameroon (AEZ5), *B. vulgaris* covers a total area of about 219,095 ha**,** which represent 18 % of total area covered by bamboo in Cameroon [9]. In Cameroon in general and in AEZ5 in particular, knowledge about the potential of *B. vulgaris* for carbon sequestration in climate change mitigation is lacking. Nevertheless, the absence of a site-specific allometric equation for *B. vulgaris* in the bimodal agroecological zone to estimate its biomass may promote the use of general equations which may result in inaccurate (biomass/carbon) estimates. Aligned with this argument, the used of the site-specific allometry equation of Nfornkah et al. [10] developed in evergreen rainforest agroecological zone is not appropriate for the bimodal rainforest agroecological zone for *B. vulgaris* biomass estimation. It appears in this sense urgent to have a site-specific allometry equation of *B. vulgaris* for AEZ5. Furthermore, existing literature reveals only the allometry equation for culm bamboo estimation. In addition, so far, no study was done to develop clump allometry although some of their parameters are relatively easy to collect e.g., number of culm and girth. As a pioneer study in this sense, it was important to investigate if model adjustment is better when considering culm or clump.

Despite having a rich bamboo resource, Cameroon has not harnessed the potential of bamboo for forest landscape restoration and climate change mitigation [15]. With the high potential of bamboo area in AEZ5 which is unsustainably managed, it appears important to evaluate their potential in terms of carbon stocks with the aim to make reliable data available to policy makers. The fast growing-rate of bamboo resulting in high carbon stocks potential (16–128 tC.ha^−1^), coupled to its inclusion in the voluntary carbon market and payment for ecosystem services can generate significant financial resources for local people to improve their livelihood [6]. Against this backdrop, this study aims to develop site-specific allometry equation for *B. vulgaris* biomass estimation and estimate their carbon stocks potential in AEZ5 of Cameroon for climate change mitigation.

## 2 Study area and methods

### 2.1 Study area

**Fig. 1:**
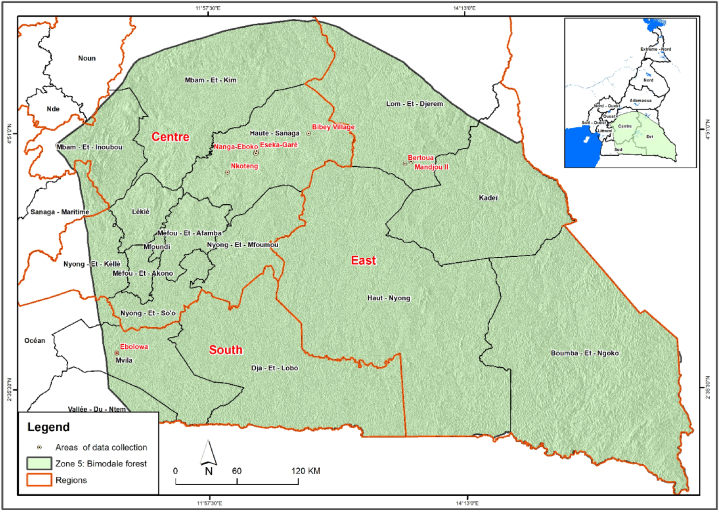
Map of the study area.

This study was carried out in bimodal rainfall forest (AEZ5) of Cameroon situated between longitude 10° 20 35 and 15° 23″ 35′ E; and latitude 5° 46” 50’ and 2° N; covering 39.9 % of the national territorial surface area of Cameroon. AEZ5 (Fig. 1) is composed of humid tropical forests, with a particularly dense hydrographic network, extending over the East (20.7 %), the Centre (12.3 %) and the South (6.4 %). The climate in this AEZ5 is characterized by four seasons of unequal duration (2 rainy seasons and 2 dry seasons). The average rainfall ranges between 1500–2000 mm per year, the mean annual temperature is 25 °C. Relief in AEZ5 varies from 400 to 1,000 m. a.s.l [24,25]. The soils are principally ferralitic soil, there is also the presence of hydromorphic soils at the banks of rivers [26]. According to Letouzey [27], AEZ5 is made up of different types of ecosystems where the forest strata of semi-deciduous and evergreen rain forests are more dominated. *B. vulgaris*, an exotic species is the dominant bamboo species in AEZ5 [12,16].

### 2.2 Methodology

#### 2.2.1 Sampling design and data collection

**Fig. 2:**
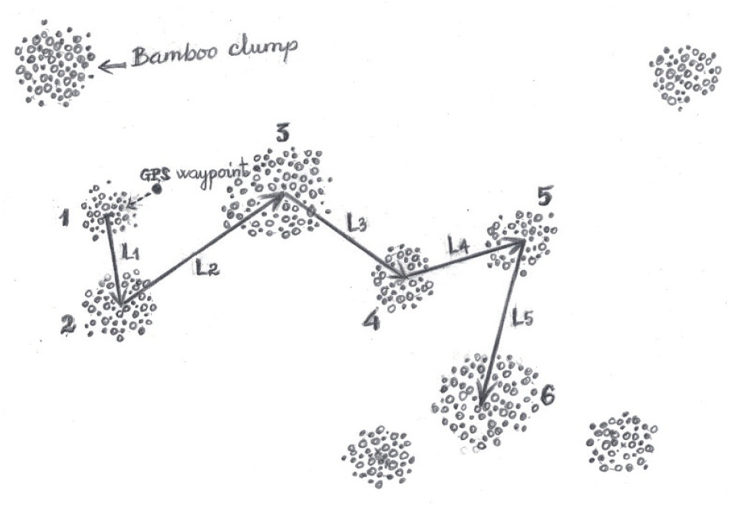
Clump-based sampling for clumping bamboo.

Data were collected in 7 administrative units (Bibey, Nanga, Nkoteng, Ebolowa, Bertoua 1 and Mandjou) which are all found in AEZ5. The sampling design used for data collection was the clump-based method as recommended by Huy and Trinth [26] for clumping bamboo. Relative to a reference point, the nearest bamboo clump was determined and from there, five distances between six nearest bamboo clumps were measured sequentially (Fig. 2). The girth (G_clump_) of each clump was measured with a graduated measuring tape. The height (H_clump_) of each clump in the plot was measured by considering the mean height of five randomly harvested bamboos culms of 1 year old, this was done for every clump. Fig. 2 represents a single plot according to this clumping-based sampling design method. In total, 20 clumping-based plots were established in the different areas for bamboo density and biomass inventory.

#### 2.2.2 Biomass data collection

**Fig. 3:**
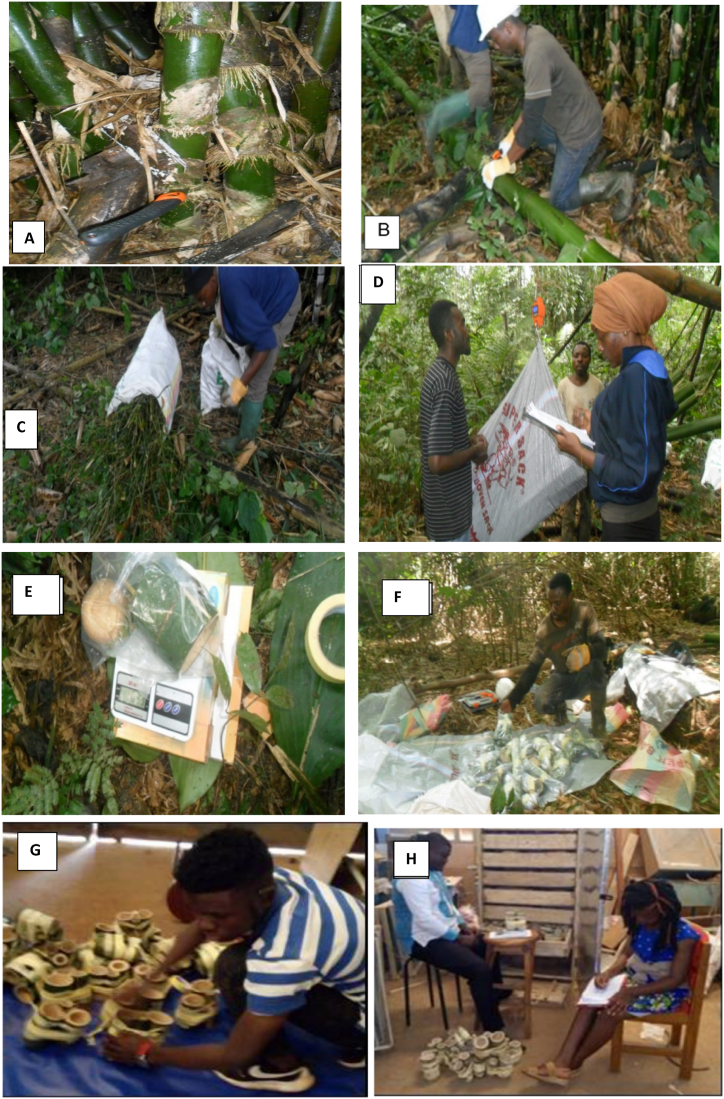
bamboo biomass data collection step by step. A: bamboo identified and harvested; B: measured and separated in to components: culm, branches and leaves; C:loaded in bags; D: weighed and recorded Fresh Weights in data sheets; E: subsamples collected and weighed with precision scale for subsample weights and recorded on data sheets; F:coded and packed in to bags and transported to laboratory; G: subsamples' codes were transferred to data sheets, prepared subsamples and dried in Oven; and H: dry weights of subsamples were recorded on data sheets.

According to the sampling design, bamboo biomass inventory for allometry equation development was done on the 3rd and 5th bamboo clumps in each plot [26]. Before felling the bamboos, G_clump_ (m) was measured at the level of the soil, average H_clump_ (m) was measured with the penta-decameter, diameter (D_clump_) was measured with the calliper and N_culm_ (number of culms per clump was counted) of each target clump were recorded. Bamboo morphological differences (First growth year of bamboo: sheath still exists on stem, usually near the roots; stem colour light green and covered by “white powder”; very few young primary branches occur on the upper stem. For year 2: absence of sheath, stem is green in colour, many branches including secondary branches, white powder persists on stem and no or minimal lichen present near roots; and for year ≥3: main stem colour is dark green, to greenish-yellow, lichens cover 30–40 % to 70–80 % on stem surface, many branches mainly on top and old branches manifest dark green with spotted lichen) were used to differentiate bamboo plants (culms) into different age groups [27,28]. Three age groups were established including 1, 2 and ≥ 3-year-old bamboos [29]. Five per cent of each age group per clump (3rd and 5th clumps) was felled for biomass sampling. For all the felled culms, diameter at 1.5 m above the soil was measured with a tree caliper. The total height of each felled culm was measured with a measuring tape. This was followed by separating the leaves and branches from the culms and their fresh biomass weight was measured using an electronic scale of 300 kg capacity. For every subsample of culms, branches and leaves, an approximate 100–300 g was collected with the help of a precision electronic scale of 0.1 g capacity. The subsamples were then labelled and conserved in adequate dry bags and transferred to the laboratory of Rural Engineering of the University of Dschang where they were oven dried at 105 °C to determine the fresh-to-dry mass ratios. Measurements were recorded in standard data sheets proposed by Huy and Trinh [26] (Fig. 3). A total of 40 clumps with a representative range of D_clump_ sizes (D_clump_ = G_culm /_π) and 86 bamboo culms were harvested from the 3rd and 5th clumps of all the 20 plots. The bamboo data collected in these 20 plots was used later to estimate biomass/carbon stocks of bamboo per hectare using site-specific allometry equations established.

### 2.3 Data analysis

#### 2.3.1 Allometric equation establishment

For the development of the allometric equations and explanatory graphs to show the log-log linear relationship existing between response variables and principal predictive biomass, R software was used. Response variables were clumping bamboo above ground biomass (AGB_clp_): Dry bamboo biomass of the culm (AGB_cl_, kg), branches (AGB_br_, kg), leaves AGB_le_, kg) and Total aboveground biomass:

Eq. (1) $${\text{AGB}}_{\text{bamboo}}={\text{AGB}}_{\text{cl}}+{\text{AGB}}_{\text{br}}+{\text{AGB}}_{\text{le}}$$

Predictive variables considered were diameter of culm (D in cm); total height of culm (H in m) and age class of culm (A in year) for culm [28,30,31] and Girth G_clump_ (m), average H_clump_ (m), diameter (D_clump_) and N_culm_ (number of culms per clump for each clump).

Several models exist as well as the power function. The power function has been widely preferred for most of the biomass allometric equations [32,33]. Yuen et al. [6] introduced the power function as an appropriate model for estimating biomass of different bamboo species. Cross-validating different model forms has often shown the power model to produce the best goodness of fit and the lowest values of statistical errors. Therefore, using power form should be encouraged for bamboo biomass allometric equations. The power form can be fit using log-transformed data linear regression [32]. Kumar et al. [30] and Yuen et al. [6] also used log transformation to fit a power model for *Bambusa nutans*. The logarithm function was used for the biomass allometric equations of *B. vulgaris* developed for this study because logarithmic transformation in most cases consider the heteroscedasticity of the data on the original scale that satisfies normality of residuals and homogeneity of variances [[32], [33], [34]]. However, the logarithmic transformation introduces bias into the model which was corrected in this study for each model established by the calculation of the correction factor:

Eq. (2) CF=RSE²2

Where, CF= Correction factor and RSE = Residual standard error.

For each response variable (leaves, branches, culms and AGB_bamboos_), several models were tested. First models tested included only one predictive variable (diameter, height, or age group), and secondly those which took into account two variables and finally models which took into account three predictive variables. For clump, the models including only one predictive variable (girth or number of culms) was tested; then those which considered two variables, followed by those which considered three predictive variables and finally, models which considered four predictive variables. The more predictive variables are added in a model, the more the model's quality improved, thereby reducing the uncertainty [10].

Important model fit statistics were used to evaluate, compare, and select the best equation [10,[34], [35], [36], [37]], those considered in this study were.

• adjusted coefficient of determination (adj.R^2^): it is used to assess the model's performance; an adj.R^2^ coefficient closer to 1 is considered better. For the comparison of two models using this statistical parameter, the best model is the model with the highest adj.R^2^ value;

Eq. (3) Adj.R2=1−1n−p∑i=1n(yi−yˆi)2/∑i−1n(yiy‾i)2

Eq. (4) Adj.R2=1−RSS/dffRSS/dft

• Akaike Information Criterion (AIC) [38] is used as a key statistic to compare and select the optimal models. The model that has the lowest AIC value is selected as the best model.

Eq. (5) AIC=−2ln(L)+2p

where L is the likelihood of the model, and p is the number of the parameters of the model.

• Residual standard error (RSE): it is a metric used to evaluate the goodness of fit of a regression model. It measures the average distance between the observed values and the predicted values. It best possible score is 0.0, smaller value is better. Range = [0, +inf).

Eq. (6) RSEy,fi=∑i=1n(yi−fi)2n−p−1

• The coefficient of variation (CV%) that correspond to the ratio between standard deviation on average of biomass for each model inside each bamboo component;
• The parameter exists if the significance of p-value <0.05.

For model validation, the parameters RRMSE (« Relative Root Mean Square Error ») and the Bias (%) were used.

Eq. (7) RRMSE=1n∑i=1n(Mpi−MiMi)2

and the

Eq. (8) Bias(%)=100×1n∑i=1n(Mpi−MiMi)

where M_pi_ is the predicted biomass « i », M_i_ is the observed or measurement « i » and n = number of sizes) in addition to test the best fit of the model, were also used for cross-validation of the model [34]. In fact, these two statistical tests for each model, allow to make a comparison of the estimated biomass and observed or measurement biomass on the field. Nevertheless, in the context of this study, the cross-validation focused on the value of the bias (%) of each model.

Furthermore, the reliability of the best model established by Nfornkah et al. [10] for *B. vulgaris* biomass estimation in evergreen rain forest of Cameroon was tested for this study area. RRMSE and Bias (%) were used to appreciate it accuracy concerning culm biomass estimation.

#### 2.3.2 Density and biomass estimations for B. vulgaris in AEZ5

*B. vulgaris density estimation:* To estimate the density of *B. vulgaris* (N_clump_.ha^−1^), the following formulae [26] were used:

Eq. (9) $$N_{clump}{ha}^{-1}=\frac{10^{4}}{{\left\{\frac{\sum_{i=1}^{5}L_{j}}{5}\right\}}^{2}}$$

Where: L_i_ represents the distance between two consecutive clumps in m.

To estimate the number *B. vulgaris* culms per ha, the following formulae was used:

Eq. (10) $$N_{i\;\text{culm}}{\text{ha}}^{-1}={\text{average}\;N}_{i\;\text{culm}}\times N_{\text{clump}}{\text{ha}}^{-1}$$

*B. vulgaris biomass estimation:* The formulae used for aboveground biomass (AGB) bamboo calculation were:

Eq. (11) $${AGB}_{bamboo}{ha}^{-1}={\bar{ABG}}_{culm}\;x\;N_{culm}x\;N_{clump}{ha}^{-1}/1000$$

Where ${\bar{ABG}}_{culm}$ is the average culm weight in kg; $N_{culm}$ is the number of the culms per ha; $N_{clump}{ha}^{-1}$ is the number of clumps per ha and AGB was given in t.ha^−1^.

*B. vulgaris carbon stocks estimation:* According to Huy and Trinh (2019), the carbon content in bamboo is estimated with Huy and Trinh [26] carbon value of 47 %. Thus, the *B. vulgaris* carbon stocks was estimated using the following formulae:

Eq. (12) $$\text{Carbon}\;\text{stock}\;\left(t\;C\;{\text{ha}}^{-1}\right)=\text{biomasses}\;\left(t\;{\text{ha}}^{-1}\right)\;x\;47\;\%$$

Conversion 1 t C = 3.67 t CO_2eq._

Eq. (13) $$\text{Stock}\;{\text{CO}}_{2}\;\left({\text{tCO}}_{2}\text{eq}\;ha^{-1}\right)=\text{Carbon}\;\text{stock}\;\left(t\;C\;{\text{ha}}^{-1}\right)\;x\;3.67$$

## 3 Results

### **3.1** Allometric equation for B. vulgaris

#### 3.1.1 Log-log linear relationship between B. vulgaris diameter and biomass components

**Fig. 4:**
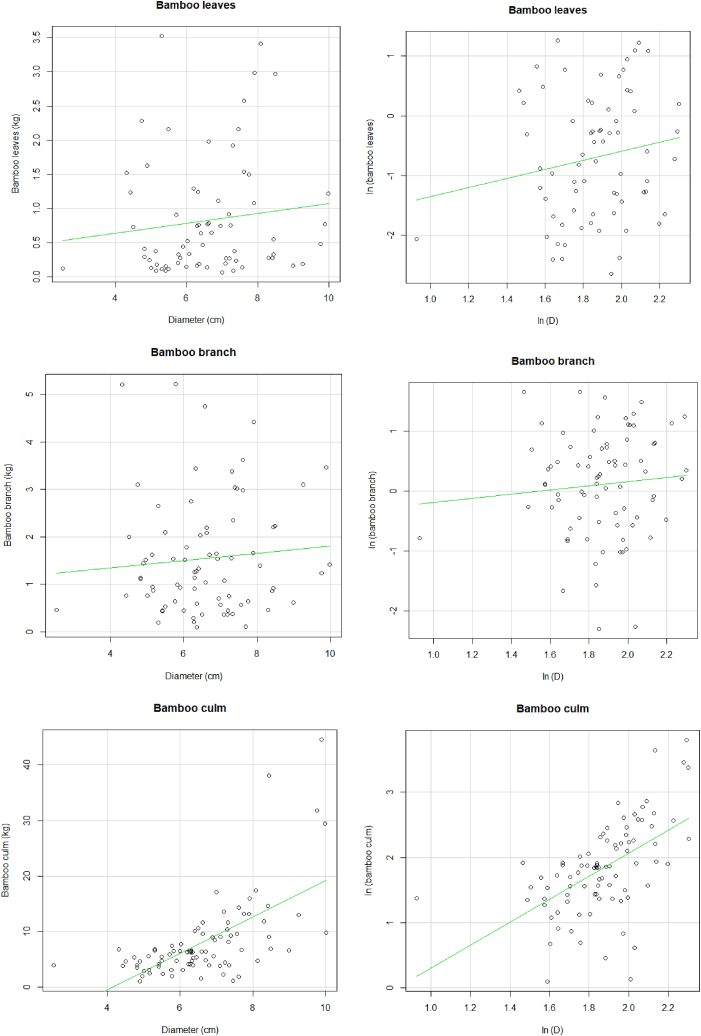

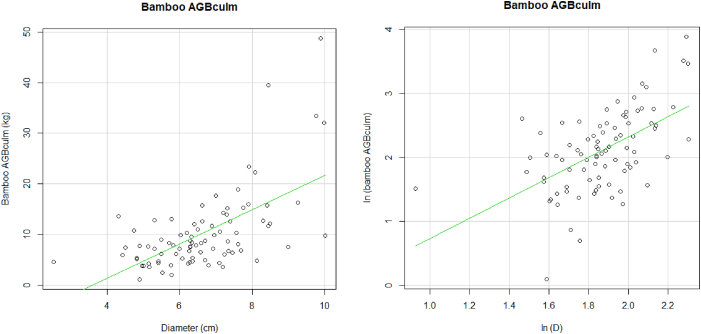
Relationship between leaves, branches, culms and AGB *B. vulgaris* biomasses of *B. vulgaris* with bamboo diameter at 1.5 m and with number of culm (N_clump_) and girth (G_clump_) for *B. vulgaris* in AEZ 5.

Fig. 4 is *B. vulgaris* biomass components in AEZ5, depicts the log - log linear relationship existing between leaves, branches, culms, and bamboo biomass with the diameter of bamboo. In general, linear regression showed an increasing bamboo biomass component with the increasing culm diameter and girth clump.

For bamboo leaves, the graph shows a weak linear relationship between diameter and leaves biomass. This was also noted for branch component. An increase in bamboo diameter shows a weak increase in branch biomass. Concerning the relationship between culm and culm diameter, the relationship was very strong, meaning an increase in culm diameter increases culm biomass. The linear relationship of diameter and all the components show a strong linear relationship. This means that, as the diameter increase, the total biomass of the bamboo increases as well.

Table 1 shows a descriptive summary of data used to develop the allometry equation of *B. vulgaris* biomass components in AEZ5 of Cameroon. Table 1 showed that culm diameter and height varied respectively from 2.53 to 10.02 cm (average = 6.62 cm) for diameter and 3.45–33.00 m for total height (average = 13.14 m). Culm AGB varied from 1.11 to 48.76 kg with an average of 10.48 kg.culm^−1^. For AGB of Clump, it varied from 50.08 to 9884.69 kg with an average of 922.41 kg.clump^−1^. Concerning biomass proportion (%) of culm components in the total aboveground of the plant (stem), the average proportions found were 77 %, 15 % and 8 % respectively, for culms, branches, and leaves biomass for the total aboveground plant.

**Table 1:** Descriptives statistics of measurements of bamboo variables.

Characteristic	N	Minimum	Maximum	Mean	Stand. dev.
For culm components					
Diameter (cm)	86	2.53	10.02	6.62	1.40
Height (m)	86	3.45	33	13.14	4.43
Age (year)	86	1	3 and more	–	–
Leaves (kg)	70	0.07	3.53	0.83	0.86
Branches (kg)	78	0.10	5.22	1.55	1.21
Culm (kg)	86	1.11	44.53	8.11	7.32
AGB culm (kg)	86	1.11	48.76	10.49	5.32
For clump					
Number of culm	40	15	346	81	75
Girth (m)	40	2.39	22.68	9.49	4.28
Average diameter (cm)	40	4.32	8.67	6.52	1.02
Average height (m)	40	7	23.86	12.64	3.31
Clump biomass (kg)	40	50.08	9884.69	922.41	1618.24

#### 3.1.2 Allometric equations for B. vulgaris biomass components

Data set of destructive biomass of *B. vulgaris* from 86 culms and 40 clumps were used for the development of culm and clump allometric equations. The results showed globally that, the consideration of all the predictive variables gave the best adjustments according to the results of statistical test for each model (AIC, RSE and Adj.R^2^).

For leaves, branches and culm biomass components, all the models obtained have Adj.R^2^ <0.49. This Adj.R^2^ varied from −0.008 to 0.133 for leaves, −0.005 to 0.346 for branches; −0.005 to 0.482 for culm. For AGB bamboo, it varied from 0.062 to 0.628 for AGB culm. These weak values of Adj.R^2^ confirmed the weak relationship between bamboo biomass components and the predictive variables. However, concerning TAGB_clump_, the Adj.R^2^ were high for these models and varied from 0.724 to 0.858.

For culm biomass components and according to statistical test done for each model (AIC, RSE and Adj.R^2^), age class appeared to be a good predictor of leaves and branches biomass as well as the height and diameter. Whereas, for culm and TAGB_culm_ biomass, it was the height that appeared as the best predictive variable than others. The consideration of the models two-by-two showed that with diameter, the addition of height improved the adjustment for culm and AGB culm. This was not the case for leaves and branches biomass components. Diameter and age class improved the adjustment better for leaves and branches than culms and TAGB_culm_ where it was instead the diameter and height that provided a good adjustment compared to diameter and age class. However, for these entire components, it was the consideration of all these three predictive variables which provided the best adjustment (Table 2).

**Table 2:** Allometric equations for *B. vulgaris* bamboo biomass components and clump estimation in agroecological zone 5 in Cameroon.

Equations	N_b_	D range	a	b	c	d	e	RRSME	RSE	Adj.R^2^	AIC	CF	Bias (%)	CV(%)	P-value
**Leaves biomass**															
lnTAGB_le_ = a + b × ln(D)	70	2.5–10.0	−2.111*	0.760^ns^				3.12	1.041	0.014	208	0.54	−174.45	16	0.162
lnTAGB_le_ = a + b × ln(H)	70	2.5–10.0	−1.366^ns^	0.265^ns^				3.21	1.052	−0.008	210	0.55	−180.30	9	0.504
lnTAGB_le_ = a + b × ln(A)	70	2.5–10.0	−1.859***	1.312**				2.90	0.982	0.122	200	0.48	−151.40	41	0.002
lnTAGB_le_ = a + b × ln(D) + c × ln(A)	70	2.5–10.0	−3.116***	0.687^ns^	1.288**			2.83	0.976	0.133	200	0.48	−148.27	42	0.003
lnTAGB_le_ = a + b × ln(D) + c × ln(H)	70	2.5–10.0	−1.969^ns^	0.911^ns^	−0.168^ns^			3.17	1.048	0.001	210	0.55	−177.07	16	0.359
lnTAGB_le_ = a + b × ln(D) + c × ln(H) + d × ln(A)	70	2.5–10.0	−3.570**	0.298**	0.424^ns^	1.410^ns^		2.87	0.979	0.128	201	0.48	−148.33	46	0.007
**Branches biomass**															
lnTAGB_br_ = a + b × ln(D)	78	2.5–10.0	−0.532^ns^	0.347^ns^				2.588	0.872	−0.005	204	0.38	−148.09	8	0.440
lnTAGB_br_ = a + b × ln(H)	78	2.5–10.0	0.046^ns^	0.027***				1.322	0.875	−0.013	205	0.38	−35.20	1	0.934
lnTAGB_br_ = a + b × ln(A)	78	2.5–10.0	−1.061***	1.433***				1.049	0.712	0.330	172	0.25	−30.74	44	<0.001
lnTAGB_br_ = a + b × ln(D) + c × ln(A)	78	2.5–10.0	−1.625*	0.304^ns^	1.430***			0.76	0.713	0.328	174	0.25	−23.11	44	<0.001
lnTAGB_br_ = a + b × ln(D) + c × ln(H)	78	2.5–10.0	−0.333^ns^	0.563^ns^	−0.237^ns^			1.03	0.876	−0.014	206	0.31	−28.20	9	<0.001
lnTAGB_br_ = a + b × ln(D) + c × ln(H) + d × ln(A))	78	2.5–10.0	−2.296**	−0.297^ns^	0.652^ns^	1.590***		0.77	0.703	0.346	172	0.25	−22.90	48	<0.001
**Culm biomass**															
lnTAGBc_l_ = a + b × ln(D)	86	2.5–10.0	−1.463**	1.764***				1.24	0.590	0.300	157	0.17	−43.97	37	<0.001
lnTAGBc_l_ = a + b × ln(H)	86	2.5–10.0	−1.640***	1.376***				1.19	0.539	0.415	141	0.15	−37.80	48	<0.001
lnTAGB_cl_ = a + b × ln(A)	86	2.5–10.0	1.725***	0.141^ns^				1.44	0.706	−0.005	188	0.25	−63.43	6	<0.001
lnTAGB_cl_ = a + b × ln(D) + c × ln(A)	86	2.5–10.0	−1.627**	1.779***	0.181^ns^			1.28	0.589	0.303	158	0.17	−44.13	38	<0.001
lnTAGB_cl_ = a + b × ln(D) + c × ln(H)	86	2.5–10.0	−2.232***	0.749*	1.056***			1.19	0.527	0.441	139	0.14	−35.93	50	<0.001
lnTAGB_cl_ = a + b × ln (D^2^ × A) + c × ln(H)	86	2.5–10.0	−2.664***	0.343***	1.171***			1.25	0.510	0.482	132	0.13	−34.30	54	<0.001
**AGB biomass**															
lnTAGB = a + b × ln(D)	86	2.5–10.0	−0.850***	1.587***				0.55	0.535	0.296	140	0.14	39.45	24	<0.001
lnTAGB = a + b × ln(H)	86	2.5–10.0	−1.144***	1.291***				0.44	0.474	0.447	120	0.11	17.16	39	<0.001
lnTAGB = a + b × ln(A)	86	2.5–10.0	1.793***	0.423*				1.29	0.617	0.062	165	0.19	−46.65	16	0.012
lnTAGB = a + b × ln(D) + c × ln(A)	86	2.5–10.0	−1.266***	1.623***	0.460***			0.84	0.503	0.376	131	0.13	−29.26	37	<0.001
lnTAGB = a + b × ln(D) + c × ln(H)	86	2.5–10.0	−1.607***	0.587^ns^	1.041***			0.63	0.466	0.466	118	0.11	−23.66	46	<0.001
lnTAGB = a + b × ln(D) + c × ln(H) + d × ln(A))	86	2.5–10.0	−2.374***	0.402^ns^	1.286***	0.652***		0.46	0.389	0.628	87	0.08	−16.06	56	<0.001
**Clump bamboo**															
lnTAGB_clp_ = a + b × ln(G)	40	2.4–22.7	1.689***	2.013***				1.14	0.582	0.724	74	0.17	−27.79	97	<0.001
lnTAGB_clp_ = a + b × ln(N)	40	2.4–22.7	0.806^ns^	1.308***				0.65	0.489	0.805	60	0.12	−25.78	128	<0.001
lnTAGB_clp_ = a + b × ln(G) + c × ln (H_av_)	40	2.4–22.7	−0.041^ns^	1.927***	0.815*			1.14	0.555	0.749	71	0.15	−38.67	124	<0.001
lnTAGB_clp_ = a + b × ln(G) + c × ln(N)	40	2.4–22.7	0.849^ns^	0.367^ns^	1.104***			0.68	0.491	0.803	61	0.12	−26.04	126	<0.001
lnTAGB_clp_ = a + b × ln(G) + c × ln (D_av_)	40	2.4–22.7	−0.748^ns^	2.016***	1.373*			1.17	0.545	0.757	70	0.15	−38.42	118	<0.001
lnTAGB_clp_ = a + b × ln(G) + c × ln(N) + d × ln (H_av_)	40	2.4–22.7	−0.630^ns^	0.313^ns^	1.058***	0.713*		0.63	0.466	0.823	58	0.11	−22.56	147	<0.001
lnTAGB_clp_ = a + b × ln(G) + c × ln(N) + d × ln (D_av_)	40	2.4–22.7	−2.275*	0.089^ns^	1.240***	1.701***		0.51	0.414	0.860	49	0.09	−18.00	153	<0.001
lnTAGB_clp_ = a +b × ln(G) + c × ln (D_av_) + d × ln (H_av_)	40	2.4–22.7	−1.158^ns^	1.944***	1.022*	0.486^ns^		1.10	0.543	0.759	70	0.15	−37.05	129	<0.001
lnTAGB_clp_ = a + b × ln(G) + c × ln (D_av_) + d × ln (H_av_) + e × ln(N)	40	2.4–22.7	−2.412*	0.098^ns^	1.550**	0.199^ns^	1.215***	0.50	0.417	0.858	50	0.09	−17.65	157	<0.001

Note: Statistical analyses are significant at 95 % confidence interval. ***p < 0.001; **p < 0.01; *p < 0.05; and ns (non-significant) p > 0.05.

AGB_clp_: Aboveground biomass of clump bamboo; AGB: Aboveground biomass of bamboo; AGB_cl_: biomass of culm; AGB_br_: biomass of branches; AGB_le_: biomass of leaves; D: Diameter of culm; H: total height of culm; A: Age of culm; G: Girth of clump (m); N: number of culm; H_av_: average height of clump; D_av_: average diameter of clump N_b_: the sample size; a, b, c, d and e are the model's fitted parameters; RRMSE: Relative root mean square error; RSE: residual standard error of the estimate; Adj R^2^: coefficient of determination; AIC: Akaike Information Criterion, CF: correction factor and CV(%): Coefficient of Variation.

For clump biomass, taking one predictive variable (Girth: G; Number of culms: N; Diameter of clump: D; and Height: H) for consideration in the model, the adjustment was better for N (Adj.R^2^ = 0.805; AIC = 60 and RSE = 0.489) compared to those with G (Adj.R^2^ = 0.724; AIC = 74 and RSE = 0.582). The comparison of the models two-by-two showed that, G-N (Adj.R^2^ = 0.803; AIC = 61 and RSE = 0.491) provided a better adjustment compared to G-D_av_ (Adj.R^2^ = 0.757; AIC = 70 and RSE = 0.545) and finally G-H_av_ (Adj.R^2^ = 0.749; AIC = 71 and RSE = 0.555). It was noted that the addition of the H_av_ in the model (G-H_av_) did not improve the adjustment. This was only the case when G and N were considered in the model. It appears not performed as models which considered only N as predictive variable which was the least. Considering three predictive variables, the inputs of G-N-D_av,_ G-N-H_av_ and G-D_av_-H_av_ models were better and when all these four predictive variables were considered in the models, the adjustment was the best (Table 2). These models gave low values of AIC, RSE, RRMSE and bias and high value of Adj.R^2^ compared to all other models tested.

Using Bias (%) for model validation for AGB biomass, the following models were validated for AGB_culm_ and AGB_clump_ biomasses estimation with the bias of −16.60 and −17.65 % respectively. However, it was also noted that all the models have negative biases (%). It was only for AGB_culm_ where two models presented a positive bias (%); thus, will provide an overestimated culm biomass. The models validated for TAGB culm and clump biomasses were:

Eq. (14) $$TAGBculm=e^{\left(-1.034+1.291\times \ln\left(H\right)\right)}\;;\;\text{Bias}=+17.16\;\%$$

Eq. (15) $$TAGBculm=e^{\left(-2.294+0.402\;x\;\ln\left(D\right)+1.286\;x\;\ln\left(H\right)+0.652\;x\;\ln\left(A\right)\right)}\;;\;\text{Bias}=-16.60\;\%$$

Eq. (16) $$TAGBclump=e^{\left(-2.322+0.098xln\left(G\right)+1.55xln\left(Dav\right)+0.199xln\left(Hav\right)+1.215\;x\;\ln\left(N\right)\right)};\;\text{Bias}=-17.65\;\%$$

The comparison of the observed data with *B. vulgaris* best model established by Nfornkah et al. (2020b) in evergreen rainfall forest showed that this model under-estimated the biomass of *B. vulgaris* in the EAZ5 where there was a bias of −45.05 % and RRMSE = 0.732. This information confirmed the necessity to have a site-specific allometric equation of *B. vulgaris* biomass estimation.

### 3.2 Bamboo density, biomass and carbon stocks per hectare of B. vulgaris

According to data inventory of *B. vulgaris* in AEZ5, average distance found between two clumps was estimated at 6.89 ± 2.2 m. The average number of culms per hectare was 20,679 ± 14,835 culm.ha^−1^; those of clumps were 257 ± 112 clump.ha^−1^ and average culm.clump^−1^ was 81 ± 75 (Table 3).

**Table 3:** Descriptive statistics of density of culm and clump, AGB culm and clump of *B. vulgaris* in AEZ 5.

Descriptive statistic	Number of plots	Mean	Min	Max	Sd
Average distance (m)	10	6.89	4.99	11.56	2.20
N_culm_ (N.ha^−1^)	10	20679	8871	57229	14835
N_clump_ (N.ha^−1^)	10	257	75	402	112
N_culm/clump_	–	81	15	346	75
Average N_culm/clump_	10	85	36	174	42
Average AGB_culm_ (kg)	10	10.30	3.54	13.50	11.37
Average AGB_clump_ (kg)	10	512.96	173.14	1177.28	354.32
AGB_bamboo_ (t.ha^−1^)	10	131.16	31.43	196.75	49.09
AGB_clump_ bamboo (t C.ha^−1^)	10	61.65	14.77	92.47	23.07
AGB_clump_ bamboo (t CO_2eq_.ha^−1^)	10	226.24	54.21	339.37	84.68

N/B**:** N_culm_ (N ha^−1^): total number of culms per ha; N_clump_ (N ha^−1^): total number of clumps per ha; N_culms clump_^−1^: number of culms per clump; mean N_culm clump_^−^: Mean number of culms per clump; Mean AGB_culm_ bamboo (kg): mean total aboveground biomass per bamboo plant (culm + branches + leaves); TAGB_clump_ bamboo (t ha^−1^): mean total aboveground biomass of bamboo per clump; TAGC_clump_ bamboo (t C ha^−1^): mean total aboveground carbon stocks of bamboo clumps per ha; TAGC_clump_ bamboo (t CO_2eq_.ha^−1^): mean total aboveground CO_2_ emission per ha.

Concerning bamboo biomass, the average culm and clump biomass were estimated respectively at 10.30 ± 11.37 and 512.96 ± 354.32 kg. In general, the bamboo average of AGB_clump_ varied from 31.43 to 196.75 t ha^−1^ with a mean of 131.16 t ha^−1^. The mean bamboo carbon stocks in zone 5 were estimated to 61.65 ± 23.07 t C ha^−1^ and 226.24 ± 84.68 t CO_2eq_.ha^−1^ (Table 3).

## 4 Discussion

### 4.1 Allometric equation for B. vulgaris

Allometric equations for this study, which were based only on AGB, were established for culm components and clump bamboos for *B. vulgaris* in AEZ5 of Cameroon. The mono-specific allometric equations was developed using a data set of 40 clumps and 86 culms. Even if *B. vulgaris* allometry equations is available for this bamboo species in evergreen rainfall forest of Cameroon, the site species-specific allometry equations is preferable for an accurate estimate of *B. vulgaris* biomass [5,39,40]. This explains why those of AEZ5 were developed.

The adjustment of the *B. vulgaris* site species-specific allometric equation for this study showed that the quality of the models varied with respect to the bamboo plant components. Xayalath et al. [40] and Nfornkah et al. [10] developed species-specific allometric equation of bamboo components of four bamboo species. They found that between leaves, branches, and predictive variables there is a weak relationship, compared to the AGB of culm which had a better relationship. Bamboo leaves and branches have limited biomass because bamboo growth in height ends in its first growing year. The variation of the diameter after the first growing season is more or less stable. Bamboo in the first year has no or few leaves and branches. These two components increase in quantity with age. This is different with trees where; their diameter keeps increasing with age while producing more branches and leaves. With trees, the increase indiameter is characterized by an increase in the number of branches and leaves, thus increasing its biomass as well. A similar result was observed in this study. For the biomass of leaves and branches, Li et al. [28]; Huy et al. [34] justify these findings by the fact that, they are response variables that are difficult to predict separately through empirical models. According to Li et al. [28], the distributions of leaf and branch biomass with bamboo diameter are dispersed and without a clear trend compared to other compartments (culm and AGB_plant_) which are better [7,34]; this is confirmed by the value of Adj.R^2^.

Compared to woody trees, the observation with diameter as predictive variable gives no satisfactory result with respect to the quality of the adjustment. According to Xayalath et al. [40], diameter only gives no good adjustment; it is the input of height into the model that improves the adjustment. However, this author did not consider the age of culm whereas age is one of the most important variables of bamboo, considering the significant morphological changes that occur with culm age [27,28]. Field observations show that 1-year old bamboo was leafless with high water content; and leaves increases with age. The older the bamboo, the greater the biomass of leaves and branches. The bamboo biomass increased with bamboo age in stands and the bamboo culm accounting for most of the total biomass [10,30,31,41]. Angom et al. [29] found that culm age structure is preponderant towards older culm age class than younger age class of bamboos in sub-tropical bamboo forest in Lengpui, North-East India.

The integration of diameter, age and height in the culm and AGB_culm_ models gave a good adjustment because these predictive variables took into account all the variability of the bamboo (culm) on the site in order to give the best estimation of their biomass. It was the same for clump biomass where the number of culm.clump^−1^, girth, average diameter and height were considered which gave a good adjustment for clump biomass estimation. Several authors found similar results according to the quality of the adjustment. It is for example the case of Melo et al. [42]; Li et al. [28]; Huy et al. [34] who found that biomass of the culm and AGB are closely related to D variable or the combination of D^2^H variables. In fact, the H-variable model has always been difficult to apply to bamboo because H is difficult to measure non-destructively, due to culm density [6,43]. Nevertheless, it is important to note that AGB adjustment that consider clump gave the best result compared to those that consider culm.

In fact, for clumping bamboo, biomass increase with the girth and the number of culms.clump^−1^. These two predictive variables are linked because for one clump of bamboos, the increasing number of culms are more correlated to the girth of the clump and biomass. During it growth, the area occupied by clump bamboo increase because more rhizomes colonize the space, and number of culms and girth increase too outwardly. This explains why the consideration of these two predictive variables in the models improved the adjustment better (Adj.R^2^ > 0.7). However, the inputs of average diameter of clumps into the models (girth +average diameter) have slightly improved the models. The variation of culm diameter in clumps is weak, reasons why 5 % of the bamboo average culm diameter were considered as predictive variable as recommended by Huy and Trinh [26]. Whereas, with the average height, the model was more improved than with average diameter. This is in concordance with finding of Xayalath et al. [40].

Because culm represents the principal factor for which the clump parameters vary, it can be concluded that, the relationship between culm or clump biomass could vary greatly for bamboo because there are parameters that have impact on bamboo species. For example, branching pattern can largely be affected by habitat conditions, such as light, clump crowding, and culm position within a clump [40].

### 4.2 Bamboo density, biomass and carbon stocks per hectare of B. vulgaris

The density of 86 clump ha^−1^ estimated by Li et al. [28] for *Bambusa stenostachya* and 184 clumps. ha^−1^ for *Oxythenanthera abyssina* in Guinea Savannah [11] were lower compared to those of this study for *B. vulgaris* which is 257 clump ha^−1^. A total of 20 clumps.ha^−1^ of *B. vulgaris* was recorded in monomodal rain forest of Cameroon by Nfornkah et al. [10]. In fact, even if these species are of sympodial category, the same or different species, the behaviour and environmental conditions and clump age may differ from one area or species to another. The average biomass of culm bamboo (*B. vulgaris*) estimated at 10.30 kg is close to those reported by other authors for bamboo species like the Moso bamboo, where culm biomass vary from 6.11 to 13.99 kg culm^−1^ [9,31]. More examples are presented in Table 4.

**Table 4:** Summarizes clump and culm densities and carbon stocks.

No	Bamboo species	Clump Density	Culm density (Culm ha^−1^)	Carbon stocks per plant (kg/plant)	Carbon stocks (t. C. ha^−1^)	References
1	*B*. *vulgaris*	257	20679	10.30	131	Kaam et al. This study
2	*B*. *stenostachya*	86			58.5 and 26.8	Li et al. [28]
3	*O*. *abyssina*	186		3.55–14.06	13.13	Nfornkah et al. [11]
4	*B*. *vulgaris*	257	2296	29	29.7	Nfornkah et al. [10]
5	*P*. *makinoi*		21191 ± 4107	6.11–13.99	105.33–49.81	Yen et al. [44]
6	*P*. *pubescens*			14	88.23 and 40.45	Zhouag et al. [31]
7	*Bambusa* sp.				30–121	Nath et al. [46]
8	*M*. *baccifera*				50.12	Angum et al. [29]
9	*B*. *tulda*				45.59	Angum et al. [29]
10	*P*. *pubescens*		4722–3400		48.31–60.58	Xu et al. [7]
11	*P*. *pubescens*				87.83–119.5	
12	*B*. sp, D. sp, G. sp, and G. sp				16–128	Yuen et al. [6]
13	*O*. *abyssinica*				10	Kaam et al. [15]

Clump age was not considered in the context of this study, even though it is a good parameter which could influence carbon stocks of bamboo. In fact, with observations during field survey, and supported by literature review of trees, girth of bamboo clump increases with increase in age of clumps [36,37,45]. For this reason, the more the girth of clump increases, the more the carbon storage increases too for bamboo species within the same environment. This observation could be explained by the high standard deviation found in this study for carbon storage per hectare of *B. vulgaris* clumps. The limitation of the girth as a predictor shall be observed with a managed bamboo forest where bamboo is sustainably harvested. The girth might not be a good predictor, however, in this case, the number of culms.clump^−1^ (N_culms._clump^−1^) will be good for biomass prediction.

However, bamboo biomass found in this study was 131 tC. ha^−1^ for *B. vulgaris*. This biomass has been compared with the biomass of different studies of the same and different bamboo species (Table 4).

This variation is because these authors considered several species for which each of them have different anatomy, belonging to different categories, and the ecological and environmental parameters of these species are different which explain the differences in carbon stocks potential. In addition, for the same species for example, several parameters can influence it carbon stocks potential including the branching pattern which can largely be affected by ecological conditions, such as light, clump crowding, and culm position within a clump [40]. Added to this is the level of management strategies for bamboo forests including the management target (for bamboo shoots or culms), and the intensity of selective cutting [21,[44], [45], [46], [47]].

The great variation in number of culms.clump^−1^ and clumps.ha^−1^ with respect to the environment of the sympodial bamboo (*B. vulgaris*), could also explain the strong variation of bamboo carbon storage found in this study. In addition, comparing research related to carbon stocks, the result of this study has demonstrated that *B. vulgaris* forest constitute one ecosystem which sequestrate an important quantity of carbon and in this sense, it contributes greatly in climate change mitigation. Thus, considering the high level of forest degradation and deforestation in AEZ5, *B. vulgaris* can be considered amongst the species that should be considered in some national reforestation and landscape restoration plans and initiatives developed by the government of Cameroon, and international initiatives ratified by the government of Cameroon. Given bamboo's fast growth rate, capacity to restore degraded landscapes, bamboo in general could be a key element to be considered by policymakers for payment of ecosystem service with a particular focus on voluntary carbon credits. Bamboo in AEZ5 is an important element within the context of climate change mitigation and adaptation. Furthermore, mindful of *B. vulgaris's* high carbon storage capacity, large distribution, and high growth rate capacity, *B. vulgaris* is a tool that the government of Cameroon can use to address some national and international strategies including the Bonn Challenge and Africa 100 million ha landscape restoration initiatives (AFR100), voluntary carbon market and more in the context of climate change mitigation in Cameroon [14,15].

This study had some limitations such as the sample size and precision. According to IPCC [48], the number of sample plots for estimating biomass and forest carbon must be determined in such a way that the error in estimation is below 10 % of the mean at each stratum at 95 % confidence level. If the error is greater than 10 %, further investigation may be needed. A preliminary inventory needs to be completed to estimate the expected variance of the biomass carbon stock in the living plants in each forest stratum. The preliminary inventory must be carried out in 10–15 (preferably 30) randomly selected plots in each forest stratum within the forest zone and/or ecological zone [49,50]. Data were collected in the bimodal rainfall forest that was too large and diverse (Fig. 1) in terms of microclimate, vegetation, soil type, topography, etc. Future investigation could be required by taking these environment variabilities to have a good representative and accurate data to make a good representation of the bamboo biomass and allometry in this area. Moreover, the study was carried out in purely natural bamboo forest, where the bamboo stands have never known management since few, or no bamboo plantations exist in Cameroon. It is also important to note that these established allometry equations are site-specific and can be used only in bimodal agro-ecological zone of Cameroon for *B. vulgaris* biomass estimation. In fact, site-specific allometric equations provide accurate and more reliable results to the site but using it in another agroecological zone can be source of important biases in estimation. The error testing analysis (a 1 % error) was not performed and should be considered in future analyses of similar studies. Similar studies are needed to determine the carbon storks and allometric equations of *Phyllostachys* sp. and *Oxynanthera abyssinica*, identified by Nfornkah et al. [9] in the AEZ5. Also, to determine effects or impacts of bamboo on soil fertility with the goal of developing bamboo-based agroforestry systems in the study area, as well as relationships between bamboo and soil water availability. These studies are critical for the development of the bamboo sector while considering ecosystem services such as food, wood, fibre, energy, medicine, (supply); and water recharge and purification, climate, air, disease (regulation).

## 5 Conclusion

This study developed *B. vulgaris* mono specific allometry equation, density, and carbon stocks in AEZ5 of Cameroon. The results of this study have shown that it is a consideration of age, diameter and height into the model which gave the best adjustment for bamboo plant (culm) component biomass; and the number of culms.clumps^−1^, girth and average diameter for the clump allometry equation. Nevertheless, model for clump biomass estimation provides better adjustment compared to those that considered culm. In other words, the comparison of the observed data with best available model showed that these model under-estimated biomass in the AEZ5, that confirm the importance of sites species specific allometry equation for *B. vulgaris* biomass estimation in a specific agroecological zone. Concerning density and carbon stocks, the results showed a high level of density per hectare in AEZ5. Carbon stocks which are estimated at 61.65 t C ha^−1^ are higher when compared to many others ecosystem in Cameroon. Bamboo forest is one of the most neglected ecosystems, which should increasingly be considered in greenhouse gas reduction strategies. This study suffered from some limitation like sample size which provides a relatively poor representation of the study area. Despite these limitations, the results can orientate policymakers and development planners in Cameroon, to capitalize on in making informed decisions in bridging the gap in carbon accounting in the forest sector of Cameroon. In addition, with the adherence of Cameroon to international instruments such as the United Nations Sustainable Development Goals (SDGs) or “the 2030 Agenda for Sustainable Development”, these findings are an additional reference that will provide substantial information to the understanding and integration of bamboo into national climate change mitigation strategies and plans as indicated by goal 13 and 15 of the UN SDGs. This is a baseline study in AEZ 5, and thus requires in-depth studies that will build on this one, considering these limitations to characterise the real bamboo carbon stocks potential in the AEZ 5 and beyond in Cameroon. Similar studies are needed on *Phyllostachys* sp. and *Oxytenanthera abyssinica*. Others should determine bamboo effect or impact on soil fertility and water recharge with the goal of developing bamboo-based agroforestry in the area.

## Data availability

Data will be made available on request.

## CRediT authorship contribution statement

**Rene Kaam:** Writing – review & editing, Writing – original draft, Funding acquisition, Formal analysis, Data curation, Conceptualization. **Martin Tchamba:** Supervision, Project administration, Conceptualization. **Barnabas Neba Nfornkah:** Writing – review & editing, Visualization, Methodology, Data curation. **Cédric Chimi Djomo:** Writing – review & editing, Methodology, Data curation.

## Declaration of competing interest

The authors declare the following financial interests/personal relationships which may be considered as potential competing interests:Rene Kaam reports financial support, administrative support, and equipment, drugs, or supplies were provided by International Network for Bamboo and Rattan. Rene Kaam reports a relationship with International Network for Bamboo and Rattan that includes: employment.
